# lncRNA UCA1 regulates miR-132/Lrrfip1 axis to promote vascular smooth muscle cell proliferation

**DOI:** 10.1515/med-2023-0738

**Published:** 2023-07-25

**Authors:** Wenming Chen, Wei Zhao, Minghui Hao, Yuping Wang

**Affiliations:** Department of Cardiology, Beijing Luhe Hospital, Capital Medical University, No. 82, Xinhua South Street, Tongzhou District, Beijing City, 101100, PR China; Department of Cardiology, Beijing Luhe Hospital, Capital Medical University, Beijing City, 101100, PR China

**Keywords:** lncRNA UCA1, atherosclerosis, miR-132, Lrrfip1, proliferation

## Abstract

UCA1 is predicted to bind to miR-132, which is a key player in the proliferation of vascular smooth muscle cells (VSMCs). This research studied the role of lncRNA UCA1 in atherosclerosis. The binding of UCA1 to miR-132 was proved by dual luciferase activity assay and RNA immunoprecipitation. UCA1 and miR-132 failed to affect each other’s expression in VSMCs. UCA1 was upregulated and miR-132 was decreased in atherosclerosis plasma. However, they are not closely correlated across atherosclerosis and control plasma sample. Interestingly, UCA1 suppressed the role of miR-132 in downregulating Lrrfip1 expression and promoting VSMC proliferation. Therefore, UCA1 is downregulated in atherosclerosis and may regulate miR-132/Lrrfip1 axis to promote VSMC proliferation.

## 1 Introduction

Atherosclerosis is a common clinical disorder of the medium- and large-sized muscular arteries caused by the formation of plaques [1]. The formation of plaque in arteries will block the supply of nutrition and oxygen to multiple important organs, such as kidney, heart, and brain, leading to a series of severe clinical disorders, such as kidney failure, heart attack, and stroke [2,3,4]. In Western counties, such as the United States, atherosclerosis is considered as the leading cause of deaths and illness [5]. In spite of the efforts made on the treatment of atherosclerosis, this disease still cannot be fully reverse, especially for the patients with advanced lesions. Therefore, novel therapeutic approaches are still needed [6].

The development and progression of atherosclerosis is a complicated process [7]. The component cells of plaque are vascular smooth muscle cells (VSMCs) [8,9]. It has been well established that the aberrant proliferation and apoptosis of VSMCs contribute to the development of atherosclerosis [8,9]. Therefore, VSMCs are promising target for atherosclerosis treatment [8]. Proliferation of VSMCs requires the participation of molecular players [1]. The sequences of lncRNAs contain no coding information, while they interact with other molecular players, such as proteins, DNAs, and miRNAs, to participate in the regulation of VSMCs [2]. FOXC2-AS1 has been reported to regulate the proliferation and apoptosis of VSMCs via regulating miR-1253/FOXF1 axis in atherosclerosis [3]. Moreover, in a recent study, Tian *et al.* reported that lncRNA UCA1 could regulate the proliferation of VSMCs by sponging miR-26a [10]. Recently, high expression levels of miR-132 during embryonic angiogenesis were reported to induce pathological angiogenesis via regulating p120RasGTPase activating protein 1, which modulates normal endothelial function [4]. Moreover, miR-132 has been reported to block VSMC proliferation and neointimal hyperplasia via regulating Lrrfip1 [5]. However, it is unclear whether UCA1-regulated miR-132 participates in atherosclerosis. Lrrfip1, or LRR binding FLII interacting protein 1, can promote the proliferation of VSMCs [11], and some miRNAs, such as miR-132, can target Lrrfip1 to suppress the proliferation of VSMCs [11]. It is not clear whether UCA1 regulates the proliferation of VSMCs through the miR-132/LRRFIP1 axis. UCA1 in this research was predicted to bind to miR-132. The crosstalk between UCA1 and miR-132 in atherosclerosis was therefore analyzed in this research.

## 2 Methods

### 2.1 Plasma samples

Blood (5 mL) was extracted from 55 atherosclerosis patients (30 males and 25 females, 24–37 years and 30.1 ± 3.8 years) and 55 healthy controls (30 males and 25 females, 24–37 years and 30.2 ± 3.9 years) under fasting conditions. All the participants were enrolled at Beijing Luhe Hospital, Capital Medical University, between March 2017 and March 2018, and informed consent was obtained. All atherosclerosis patients were diagnosed by blood test to check the level of cholesterol and Doppler ultrasound to check blockage in arteries. All healthy controls showed normal physiological functions. Blood samples were used to prepare plasma samples, which were stored in liquid nitrogen before use. The demographic and clinicopathological characteristics of the patients were recorded and are listed in Table 1.

**Table 1:** Baseline demographic characteristics between two groups

Parameter	Atherosclerosis group (*n* = 55)	Control group (*n* = 55)	*p* values
Age	30.1 ± 3.8	30.2 ± 3.9	0.789
Sex (female/male)	25/30	25/30	0.689
Smoking	32	34	0.812
Drinking	41	40	0.855
Hypertension	41	12	0.004
Diabetes	46	6	0.001
TG (mmol/L)	1.73 ± 0.26	1.62 ± 0.31	0.321
TC (mmol/L)	5.20 ± 0.87	4.37 ± 0.76	0.003
HDL-C (mmol/L)	1.16 ± 0.25	1.30 ± 0.33	0.031
LDL-C (mmol/L)	2.82 ± 0.63	2.41 ± 0.11	0.024

Data are reported as means ± SD. *p* < 0.05 was considered significant. TG: Triglyceride; TC: Total cholesterol; HDL-C: Fasting high-density lipoprotein cholesterol; LDL-C: Fasting low-density lipoprotein cholesterol.


**Ethics approval:** This study was approved by the Ethics Committee of the Beijing Luhe Hospital, Capital Medical University. The research has been carried out in accordance with the World Medical Association Declaration of Helsinki. All patients and control volunteers provided written informed consent prior to their inclusion within the study.

### 2.2 Human aortic smooth muscle cells (HAOSMCs) and lipofectamine 2000-mediated cell transfection

HAOSMCs (354-05A; Sigma-Aldrich, St. Louis, MO, USA) were used in this study. HAOSMCs were cultivated until about 80% confluence to perform the following transfection. To perform the overexpression experiments, UCA1 and Lrrfip1 expression vectors were constructed with pcDNA3.1. Negative control (NC) miRNA (5′-UGUUGUACGUAGUGUCGAUCCCA-3′) and miR-132 mimic (5′- UAACAGUCUACAGCCAUGGUCG-3′) were purchased from Sigma-Aldrich (USA). HAOSMCs were transfected with UCA1 or Lrrfip1 expression vector (10 nM) or miR-132 mimic (40 nM) using lipofectamine 2000 (Invitrogen, Shanghai, China). To perform dual luciferase activity assay, pGL3 vector (Promega) was used to construct UCA1 luciferase vector. HAOSMCs were co-transfected with UCA1 vector + miR-132 mimic (miR-132 group) or UCA1 vector + NC miRNA (NC group). Cells were harvested at 48 h post-transfection for the confirmation of overexpression or the measurement of luciferase activity.

### 2.3 RNA preparations and RT-qPCR

Trizol reagent (Invitrogen, Shanghai, China) was mixed with plasma or HAOSMCs to extract total RNAs. All steps were performed according to the instructions from Invitrogen, and 85% ethanol was used to precipitate RNA samples to harvest miRNAs. Two all-in-one kits from Genecopoeia (Guangzhou, China), namely, BlazeTaq™ One-Step SYBR Green real-time quantitative polymerase chain reaction (RT-qPCR) Kit (for UCA1 and Lrrfip1 mRNA) and All-in-One™ miRNA qRT-PCR Reagent Kit (for miR-132), were used to measure the expression levels of each gene using GAPDH and U6 endogenous controls. Each polymerase chain reaction was repeated three times, and gene expression levels were normalized to endogenous controls using the 2^−ΔΔCT^ method. Primer sequences were as follows: 5′-TTTGCCAGCCTCAGCTTAAT-3′ (forward); 5′-TTGTCCCCATTTTCCATCAT-3′ (reverse) for UCA1; 5′-ATGAGTTAAAGGACCAGATT-3′ (forward); 5′-TCAACCTGGTACATGAAGTT-3′ (reverse) for UCA1; 5′-GTCTCCTCTGACTTCA-3′ (forward); 5′-CCACCCTGTTGCTGTA-3′ (reverse) for GAPDH. The forward primer of miR-132 was 5′- TAACAGTCTACAGCCATG-3′. The forward primer of U6 and the universal reverse primer were used from the kit.

### 2.4 Western blot

Radioimmune precipitation assay solution (Invitrogen, Shanghai, China) was mixed with HAOSMCs to prepare protein samples, which were quantified using bicinchoninic acid assay (Invitrogen, Shanghai, China). Protein samples were incubated at 95°C for 10 min to reach protein denaturation. After electrophoresis (10% sodium dodecyl sulfate-polyacrylamide gel electrophoresis gel), gel transfer, and blocking, incubation with rabbit primary antibodies of Lrrfip1 (ab103148, Abcam, Cambridge, UK) and GAPDH (ab9485, Abcam, Cambridge, UK) and secondary antibody of HRP Goat Anti-Rabbit (IgG) (ab97051, Abcam, Cambridge, UK) was performed. ECL™ Detection Reagent (Sigma-Aldrich, St. Louis, MO, USA) was used to produce signals, which were normalized using Quantity One software.

### 2.5 Cell proliferation analysis

HAOSMCs were harvested at 48 h post-transfection to analyze cell proliferation. HAOSMCs were cultivated (3,000 cells in 0.1 mL medium per well of 96-well plates). Cells were cultivated at 37°C and were collected every 24 h until 96 h. CCK-8 solution was added into medium to reach 10% at 4 h before cell collection. Optical densities values at 450 nM were measured to reflect cell proliferation.

### 2.6 RIP

Magna RIP™ RNA-Binding Protein Immunoprecipitation Kit (Millipore) and anti-Argonaute2 (anti-Ago2) (ab32381, Abcam) or anti-IgG antibody (ab133470, Abcam) [6] were used to further validate the binding of UCA1 to miR-132. Briefly, cell lysate was co-incubated with A/G magnetic beads together with anti-Ago2 or anti-IgG antibody. After proteinase K digestion, the enrichment of UCA1 and miR-132 was detected by RT-qPCR assay.

### 2.7 Colony formation assay

Transfected cells were digested, counted, and cultured in a 12-well plate of 100 cells/well at 37℃ under 5% CO_2_ for 3 weeks. The cells were soaked in Dulbecco’s phosphate-buffered brine for two times, and 1 mL of methanol was added to each well for fixation for 15 min, and then 1 mL of Giemsa was added. Colony formation rate was assessed [7].

### 2.8 Dual-luciferase reporter assays

The potential binding site of miR-132 on UCA1 was cloned into pmirGLO-dual-luciferase reporter. UCA1 was co-transfected into cells with miR-132 or miR-132 (mut). The luminescent signals of Renilla firefly were calculated following the protocol provided in the double luciferase assay system (Promega) at 48 h after transfection.

### 2.9 Statistical analysis

Unpaired *t* test (two groups) and ANOVA Tukey’s test (multiple groups) were used to compare datasets. *p* < 0.05 was considered to be statistically significant.

## 3 Results

### 3.1 UCA1 and miR-132 can directly interact with each other

**Figure 1:**
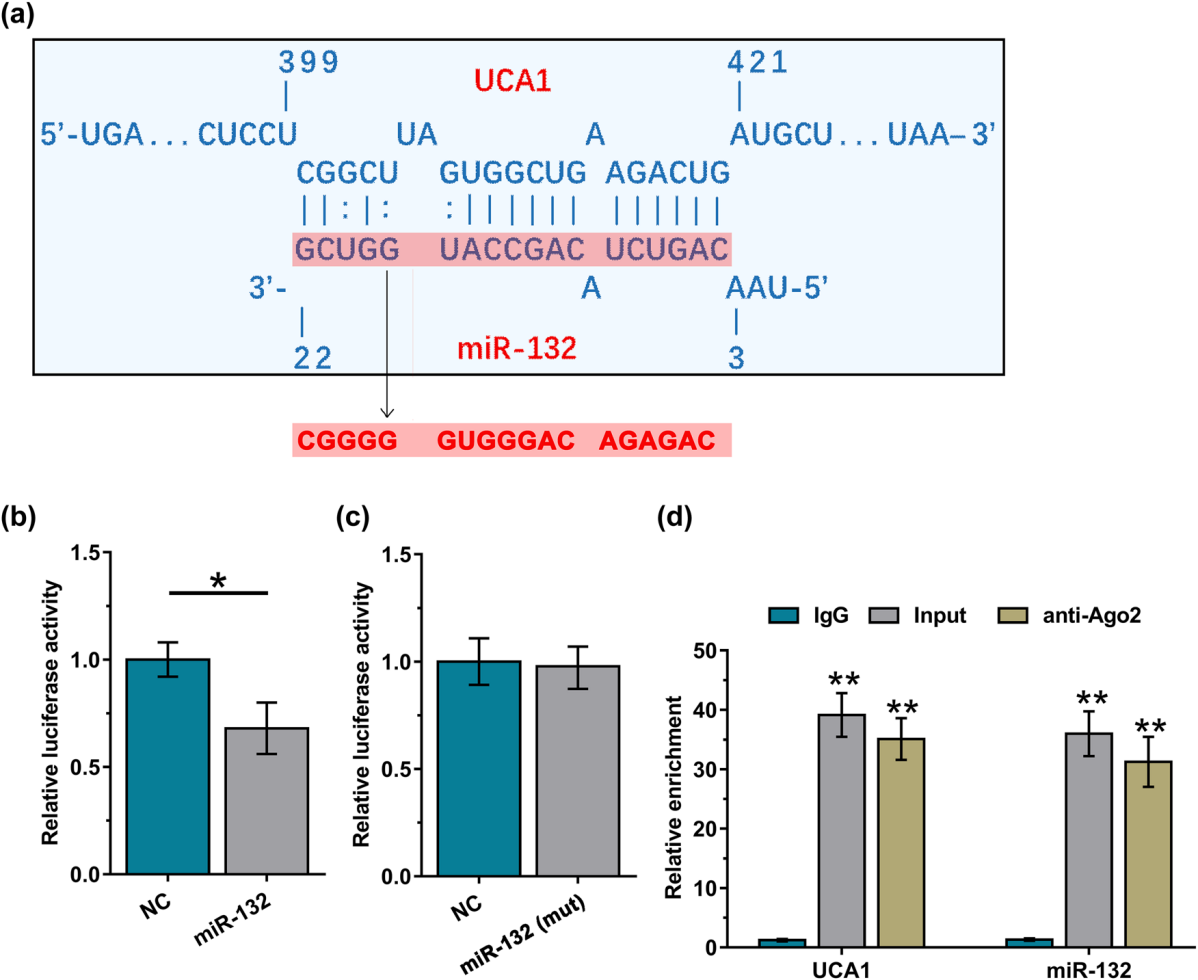
UCA1 and miR-132 can directly interact with each other. The binding of UCA1 to miR-132 was predicted using IntaRNA (a). Dual luciferase activity assay was conducted to further confirm the interaction between them (b). Dual luciferase activity was repeated using miR-132 mutant (miR-132(mut)). It was observed that miR-132 (mut) failed to reduce luciferase activity compared to NC group (c). RIP results suggested that UCA1 and miR-132 were concentrated in both anti‐Ago2 group and input group (d). *, *p* < 0.05.

The binding of UCA1 to miR-132 was predicted with IntaRNA 2.0 [12], which showed that UCA1 could bind to miR-132 (Figure 1a). The binding of UCA1 to miR-132 was further confirmed by dual luciferase activity assay. Compared to the NC group, the miR-132 group showed a decreased luciferase activity (Figure 1b, *p* < 0.05). miR-132 mutant (miR-132(mut)) was designed (indicated by letters in red, Figure 1a). Dual luciferase activity was repeated using miR-132(mut). It was observed that miR-132 (mut) failed to reduce luciferase activity compared to the NC group (Figure 1c). Moreover, RNA immunoprecipitation (RIP) results (Figure 1d) suggested that UCA1 and miR-132 were accumulated in both input and anti‐Ago2 groups as compared to the anti-lgG group. Therefore, miR-132 and UCA1 may interact with each other.

### 3.2 Regulatory role of UCA1 and miR-132 in each other’s expression

**Figure 2:**
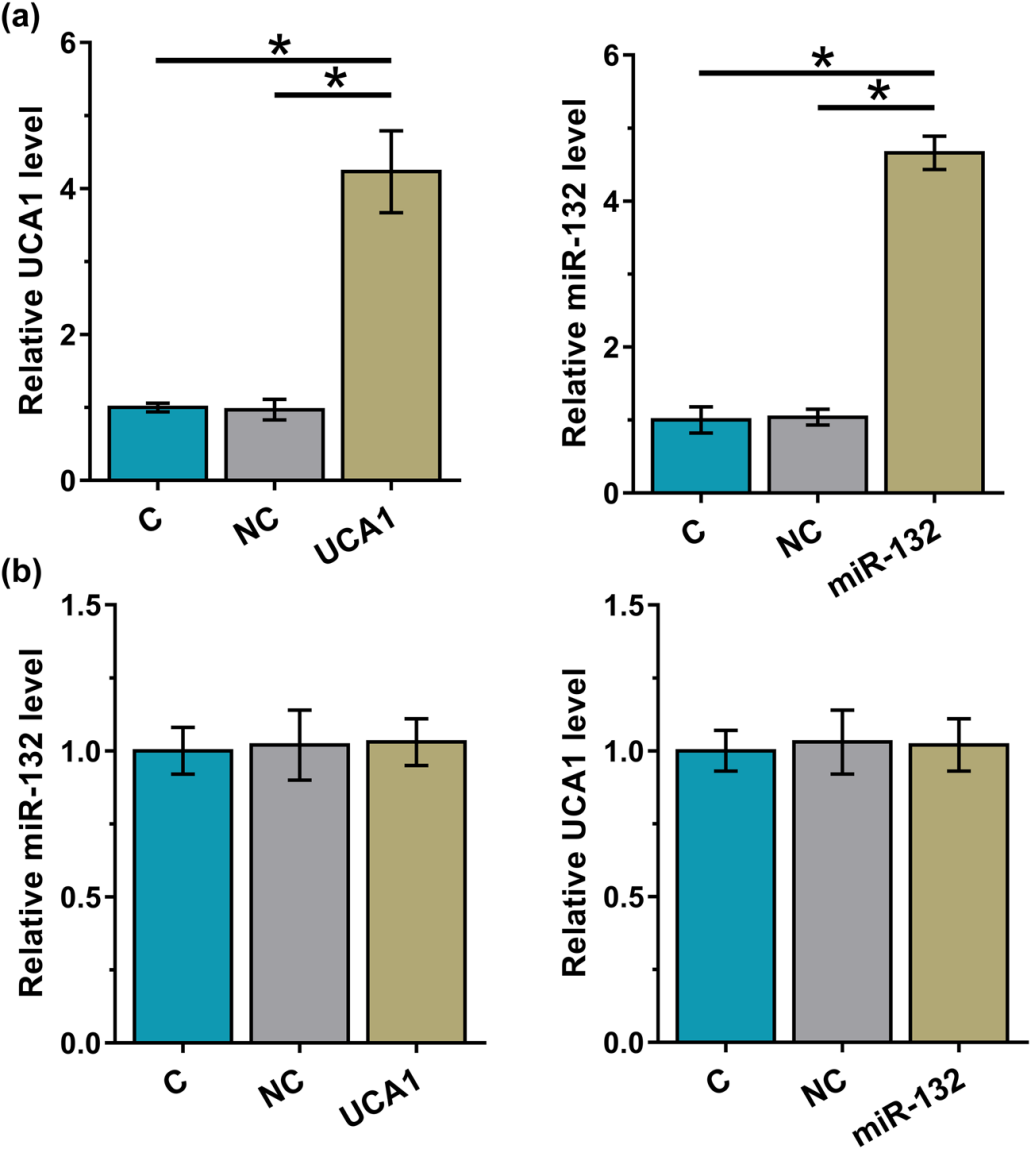
Regulatory role of UCA1 and miR-132 in each other’s expression. HAOSMCs were overexpressed with UCA1 or miR-132 (a). The effects of UCA1 and miR-132 overexpression on the expression of each other were analyzed by performing RT-qPCR at 48 h post-transfection (b). *, *p* < 0.05.

HAOSMCs were overexpressed with UCA1 or miR-132 (Figure 2a, *p* < 0.05). miR-132 failed to affect UCA1 expression. Similarly, overexpression of UCA1 did not affect the expression of miR-132 (Figure 2b, *p* < 0.05). Therefore, UCA1 may not be a target of miR-132.

### 3.3 Plasma levels of UCA1 and miR-132 were altered in atherosclerosis patients

**Figure 3:**
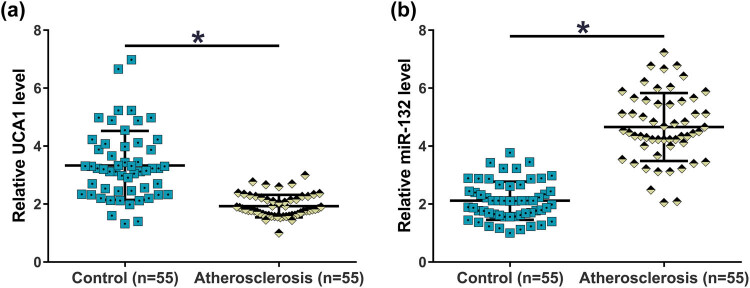
Plasma levels of UCA1 and miR-132 were altered in atherosclerosis patients. Levels of UCA1 (a) and miR-132 (b) in plasma from atherosclerosis patients (*n* = 55) and healthy controls (*n* = 55) were measured by performing RT-qPCR. *, *p* < 0.05.

UCA1 was accumulated to high amounts (Figure 3a) in atherosclerosis plasma, while miR-132 was accumulated to low amounts (Figure 3b) in atherosclerosis plasma (*p* < 0.05). These data suggest the involvement of UCA1 and miR-132 in atherosclerosis.

### 3.4 UCA1 and miR-132 were not significantly correlated

**Figure 4:**
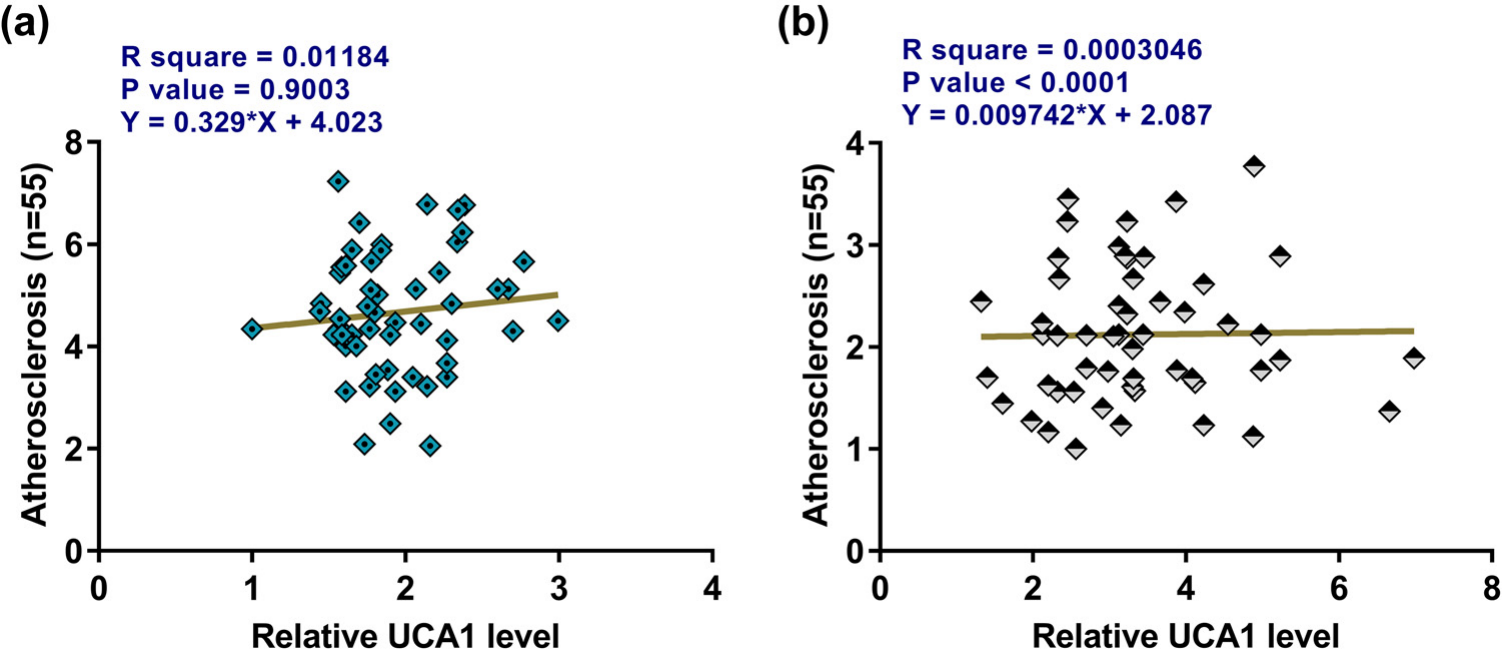
Plasma levels of UCA1 and miR-132 were not significantly correlated. Correlations between plasma levels of UCA1 and miR-132 across atherosclerosis (a) and control (b) plasma samples.

The correlations between plasma levels of UCA1 and miR-132 across atherosclerosis and control plasma samples were analyzed. No significant correlation between plasma levels of UCA1 and miR-132 was found across plasma samples from atherosclerosis patients (Figure 4a) and healthy controls (Figure 4b). Therefore, UCA1 and miR-132 may not regulate the expression of each other in the human body.

### 3.5 UCA1 regulated Lrrfip1 expression though miR-132 to promote the proliferation of HAOSMCs

**Figure 5:**
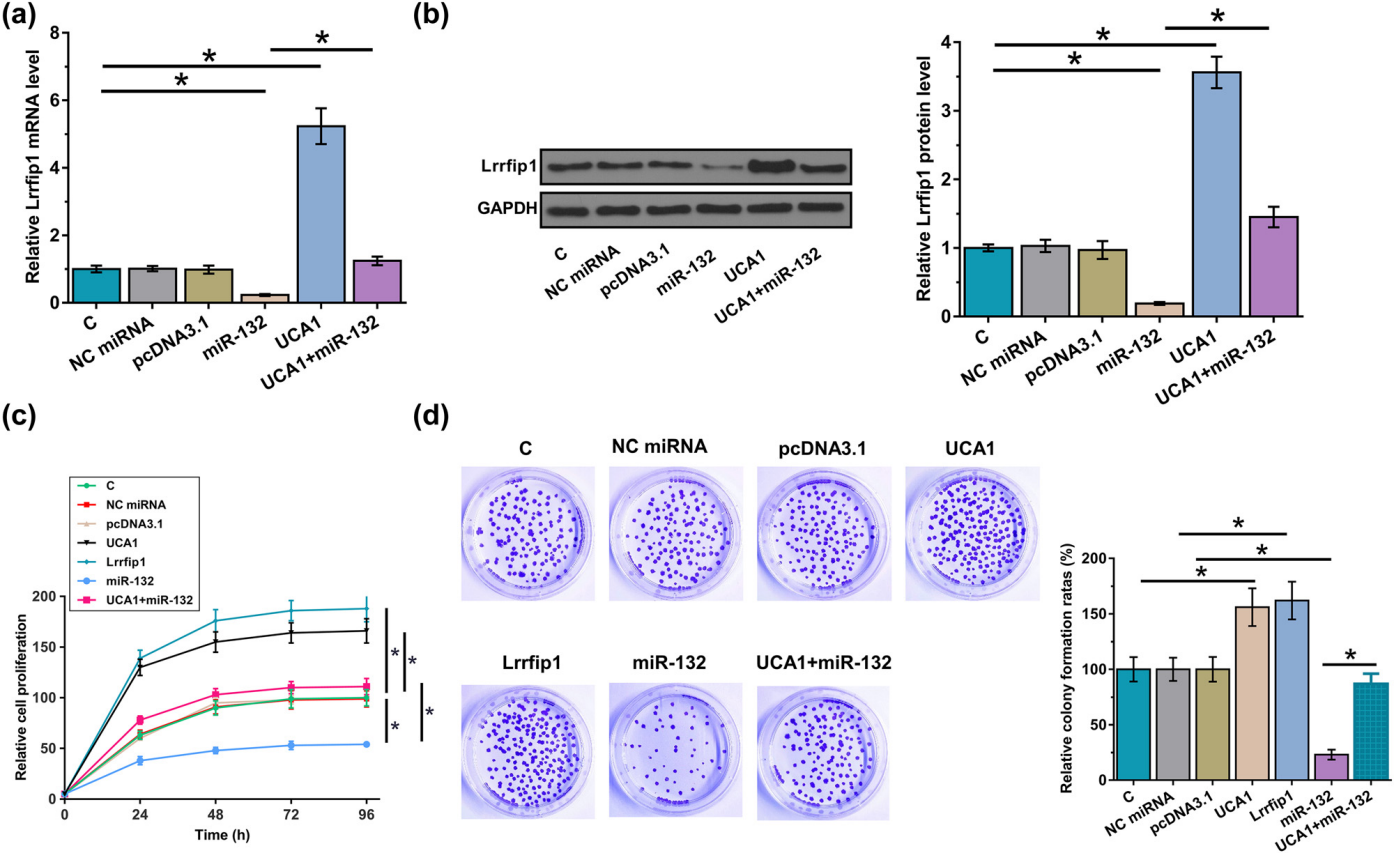
UCA1 regulated Lrrfip1 expression though miR-132 to promote the proliferation of HAOSMCs. The aforementioned data suggest UCA1 as an endogenous sponge of miR-132. To test this hypothesis, the effects of UCA1 and miR-132 overexpression on the expression of Lrrfip1, a direct target of miR-132, were analyzed by RT-qPCR (a) and western blot (b). Cell proliferation assay was performed to analyze the effects of UCA1, miR-132, and Lrrfip1 overexpression on the proliferation of HAOSMCs (c and d). *, *p* < 0.05.

The aforementioned data suggested UCA1 as an endogenous sponge of miR-132. To this end, the role of UCA1 and miR-132 in regulating the expression of Lrrfip1, a direct target of miR-132, was analyzed by RT-qPCR (Figure 5a) and Western blot (Figure 5b). UCA1 overexpression suppressed the role of miR-21 in downregulating Lrrfip1 expression (*p* < 0.05). Original Western blot images are presented in Figure S1. Cell proliferation analysis was conducted to explore the role of UCA1, miR-132, and Lrrfip1 in HAOSMC proliferation. Compared to the C group, UCA1 and Lrrfip1 overexpression resulted in increased cell proliferation rate (Figure 5c, *p* < 0.05). In contrast, miR-132 overexpression played an opposite role (*p* < 0.05). Moreover, UCA1 overexpression reversed the effects of miR-132 overexpression on the proliferation of HAOSMCs (*p* < 0.05). Furthermore, colony formation assay indicated that the overexpression of UCA1 and Lrrfip1 promoted the colony formation and the overexpression of miR-132 suppressed the colony formation. Moreover, miR-132 overexpression could counteract the simulative effect of UCA1-induced on HAOSMC proliferation (Figure 5d, *p* < 0.05).

## 4 Discussion

This study mainly investigated the involvement of the interaction between UCA1, miR-132, and Lrrfip1 in the proliferation of HAOSMCs. We found that UCA1 was increased in atherosclerosis and may upregulate Lrrfip1 by sponging miR-132 to promote the proliferation of VSMCs.

VSMCs participate in multiple processes of the development and progression of atherosclerosis [13]. In early-stage plaque, the accelerated proliferation of VSMCs promotes the plaque formation [13]. In contrast, VSMCs in advanced-stage plaques generate extracellular matrix to induce the formation of fibrous cap to stabilize plaques, thereby preventing plaque ruptures [13]. Therefore, inhibiting VSMCs proliferation in early-stage plaque and promoting their proliferation in advanced-stage plaques may assist the treatment of atherosclerosis. A considerable number of molecular players have been identified in the proliferation of VSMCs, while their involvement in atherosclerosis is unknown. In a recent study, Choe *et al.* reported that miR-132 could target Lrrfip1 to inhibit VSMC proliferation in neointimal hyperplasia [14]. In this study, we observed the downregulation of miR-132 in atherosclerosis. In another study, Das *et al.* found that silencing of UCA1 released miR-26a and led to the reduced proliferation rate of VSMCs [11]. Consistently, we reported that UCA1 overexpression led to the increased proliferation rate of VSMCs.

Interestingly, our study showed the direct interaction between UCA1 and miR-132 in VSMCs. However, overexpression of UCA1 and miR-132 failed to affect the expression of each other. In addition, although UCA1 and miR-132 showed opposite expression patterns in atherosclerosis, they are not significantly correlated with each other across plasma samples. Our data suggested that UCA1 was unlikely a target of miR-132. However, we provided sufficient evidence to show that UCA1 and miR-132 can interact with each other. Besides being the target of miRNAs, lncRNAs may also serve as the sponge of miRNAs to suppress their functions without affecting the expression of miRNAs [15,16]. Indeed, UCA1 reduced the inhibitory effects of miR-132 on the expression of Lrrfip1 and its enhancing effects on the proliferation of VSMCs. Therefore, UCA1 is likely an endogenous competing RNA of miR-132. miR-132 expression in this study was detected using RT-qPCR, in which the high temperature will release miR-132 from UCA1. Therefore, RT-qPCR analysis revealed no role of UCA1 and miR132 in each other’s expression.

Our data suggested that the fine regulation of VSMC proliferation may serve as a target for atherosclerosis treatment. Moreover, the expression of biomarkers (UCA1, miR-132) corresponding to blood samples collected was not affected by plaque stage. However, the expression of miR132 and UCA1 in atherosclerotic tissue is unclear. Atherosclerosis and control groups in this study exhibited significantly different status of hypertension and diabetes, while hypertension, diabetes, and atherosclerosis are known to be closely associated [1,2,3,4,5]. In addition, UCA1 can also affect other miRNAs to regulate the proliferation rate of VSMCs [11]. Therefore, our conclusions require further validation.

## 5 Conclusion

**Figure 6:**
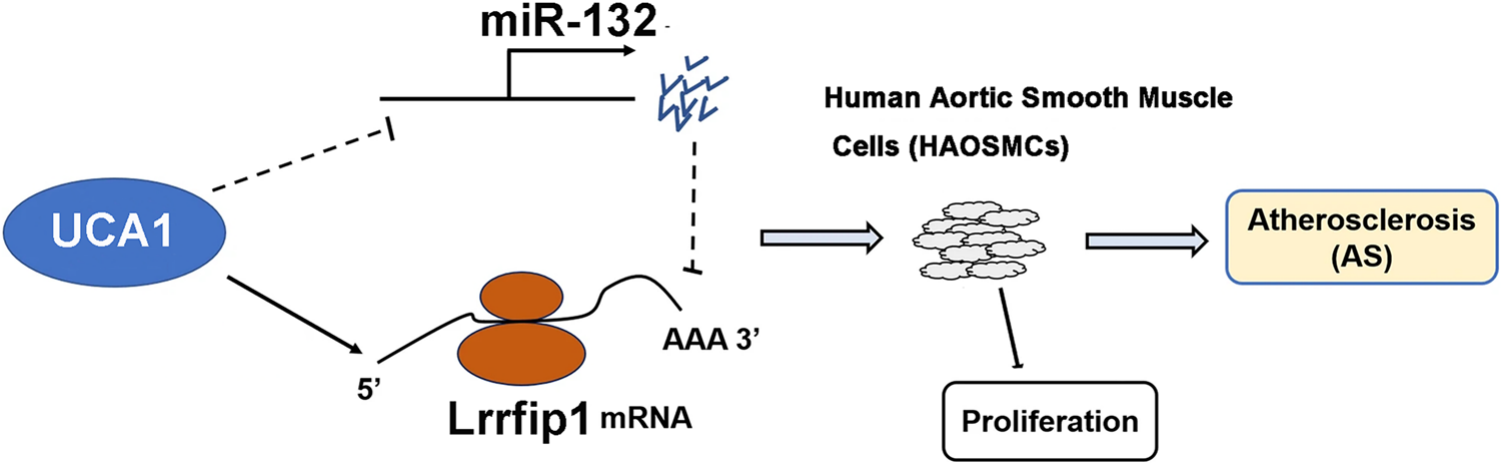
UCA1 promoted HAOSMC proliferation via the regulation of miR-132/Lrrfip1. lncRNA UCA1 may compete with miR-132 by regulating Lrrfip1 to participate in the proliferation of HAOSMCs, which suggest its critical roles in the pathogenesis of atherosclerosis.

In conclusion, UCA1 is upregulated in atherosclerosis and it may sponge miR-132 to upregulate Lrrfip1, thereby promoting the proliferation of VSMCs (Figure 6).

### List of abbreviations

HAOSMCs: human aortic smooth muscle cells
NC: negative control
VSMCs: Vascular smooth muscle cells

## Supplementary Material

Supplementary Figure
