# Supplementary material for: lncRNA UCA1 regulates miR-132/Lrrfip1 axis to promote vascular smooth muscle cell proliferation
Source: Open Med (Wars). 2023 Jul 25;18(1):20230738. doi: 10.1515/med-2023-0738 (PMC10390752; doi:10.1515/med-2023-0738)
Supplement: Supplementary Figure [file med-2023-0738-sm.pdf]

Supplementary material

Original image for GAPDH

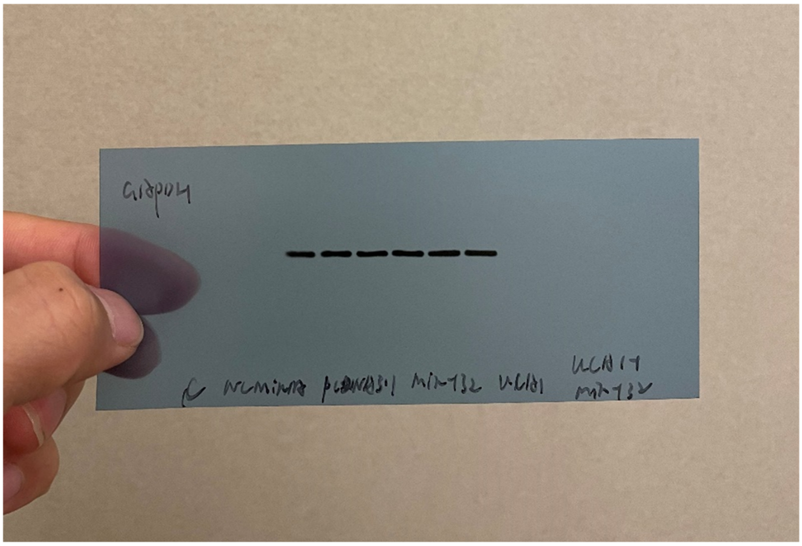

Original image for Lrrfip1

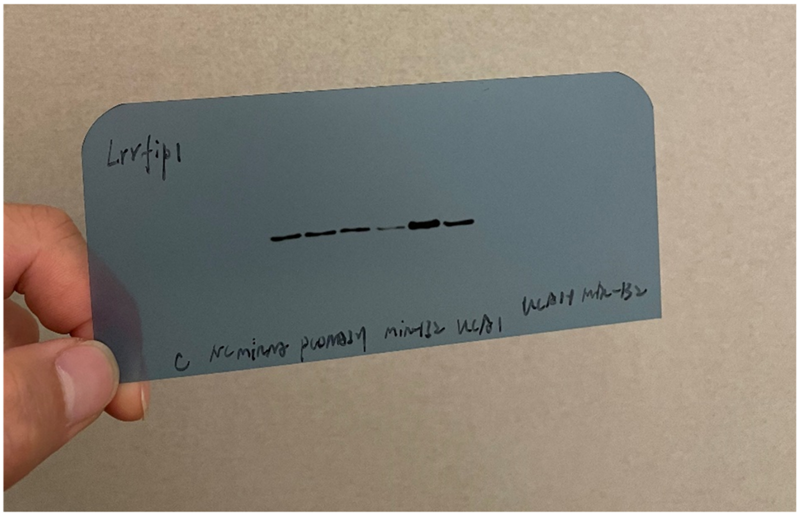

Figure S1: Original Western blot images.
